# Frequency and genotyping of group A rotavirus among Egyptian children with acute gastroenteritis: a hospital-based cross-sectional study

**DOI:** 10.1186/s12985-024-02495-8

**Published:** 2024-09-30

**Authors:** Ensaf A. Azzazy, Rania M. Amer, Ghada Mohammed Abdellatif, Hala Adel Abd-Elmoneim, Doaa Alhussein Abo-Alella

**Affiliations:** 1 Medical Microbiology and Immunology Department, Faculty of Medicine, Zagazig University, Elsharkia Governorate, Zagazig, Egypt; 2 Pediatrics Department, Faculty of Medicine, Zagazig University, Elsharkia Governorate, Zagazig, Egypt

**Keywords:** Rotavirus, Diarrhea, Genotypes, RT-PCR, ELISA

## Abstract

**Background:**

This hospital-based cross-sectional study aims to investigate the epidemiologic and clinical characteristics of rotavirus group A (RVA) infection among children with acute gastroenteritis and to detect the most common G and P genotypes in Egypt.

**Methods:**

A total of 92 stool samples were collected from children under five who were diagnosed with acute gastroenteritis. RVA in stool samples was identified using ELISA and nested RT-PCR. Common G and P genotypes were identified utilizing multiplex nested RT-PCR assays.

**Results:**

RVA was detected at a rate of 24% (22 /92) using ELISA and 26.1% (24 /92) using VP6 nested RT-PCR. The ELISA test demonstrated diagnostic sensitivity, specificity, and accuracy of 91.7%, 100%, and 97.8%, respectively. G3 was the most prevalent G type (37.5%), followed by G1 (12.5%), whereas the most commonly detected P type were P[8] (41.7%) and P[6] (8.2%). RVA-positive samples were significantly associated with younger aged children (*p* = 0.026), and bottle-fed (*p* = 0.033) children. In addition, RVA-positive samples were more common during cooler seasons (*p* = 0.0001). Children with rotaviral gastroenteritis had significantly more frequent episodes of diarrhea (10.87 ± 3.63 times/day) and vomiting (8.79 ± 3.57 times/day) per day (*p* = 0.013 and *p* = 0.011, respectively). Moreover, they had a more severe Vesikari clinical score (*p* = 0.049).

**Conclusion:**

RVA is a prevalent cause of acute gastroenteritis among Egyptian children in our locality. The discovery of various RVA genotypes in the local population, as well as the identification of common G and P untypeable strains, highlights the significance of implementing the rotavirus vaccine in Egyptian national immunization programs accompanied by continuous monitoring of strains.

**Supplementary Information:**

The online version contains supplementary material available at 10.1186/s12985-024-02495-8.

## Introduction

Diarrhea poses a significant health burden, particularly in developing countries, accounting for approximately 525,000 mortalities among children under the age of 5 annually. Rotavirus is a common cause of severe acute diarrhea in early childhood, responsible for 35–60% of cases. It manifests as vomiting, watery diarrhea, and significant dehydration. Among children seeking medical care in developing countries, the case-fatality rate of rotavirus infection is approximately 2.5% [1, 2].

The rotavirus belongs to the *Reoviridae* family and is a non-enveloped, icosahedral virus with a double-stranded RNA genome. The virus contains three protein shells that enclose its genome. The genome entails 11 double-stranded RNA segments coding six structural proteins (VP1-4, VP6, and VP7) and six nonstructural proteins (NSP1-6) [3].

Rotaviruses are classified based on the VP6 protein present in their inner capsid, leading to nine classifications ranging from A to I. RVA from group A is responsible for childhood diarrhea. Another classification is based on the outer capsid proteins VP7 (glycoprotein) and VP4 (protease-sensitive), which allow for the identification of different G and P genotypes, respectively. Currently, a minimum of 42 G and 58 P rotavirus genotypes have been documented. The most common rotavirus genotypes worldwide are G1, G2, G3, and G4, frequently associated with P[4], P[6], and P[8] [4].

Several techniques are utilized to identify rotavirus in fecal samples. These include electron microscopy, enzyme immune assay, latex agglutination test or lateral flow immune assay, polyacrylamide gel electrophoresis, and reverse transcriptase polymerase chain reaction (RT-PCR) [3]. Antigen detection techniques are the most commonly applied methods for rotavirus detection due to their relative speed, sensitivity, and specificity [5]. RT-PCR is utilized to detect rotavirus in fecal samples with higher sensitivity and specificity and to characterize G and P genotypes [6].

The prevalence of rotavirus in Egypt varied significantly across different studies conducted in Egypt, ranging from 11 to 76.9% [7]. This variation can be attributed to several factors, including geographical region and season of sampling, sociodemographic characteristics (e.g., age, sex, and type of feeding), and the specificity and sensitivity of the diagnostic methods [7, 8].

The aim of this study was to investigate the epidemiologic and clinical features of RVA infection among children with acute gastroenteritis. Furthermore, genotyping for RVA was performed to detect the most common G and P genotypes in our locality, hoping to guide decision-makers in designing future public healthcare strategies.

## Materials and methods

### Study design and ethical approval

This hospital-based cross-sectional study was carried out at the Medical Microbiology and Immunology Department and Pediatrics Department in collaboration with the Scientific and Medical Research Center, Faculty of Medicine Zagazig University, during the period between December 2021 and December 2023. Samples were collected from the Pediatrics department (Zagazig University Pediatrics Hospital), ELISA screening was performed in the laboratory of the Medical Microbiology and Immunology Department while molecular testing was carried out at the Molecular Biology laboratory of the Scientific and Medical Research Center. Approval was taken from Institutional Review Board (IRP) no. 9272. Informed written consents were assigned by the guardians of all pediatric patients included in this study.

### Sample collection

A total of 92 stool samples were collected from children under the age of 5 admitted to Zagazig University Pediatrics Hospital with acute gastroenteritis. Patients with chronic or persistent diarrhea for more than two weeks were abandoned from the study. Full medical history was collected, and a complete clinical examination was performed to obtain the following data: Patient’s name, age, sex, feeding pattern, and symptoms or signs of gastroenteritis, including fever, abdominal pain, diarrhea, and vomiting (frequency, duration, and character). Vesikari Clinical Severity score was determined based on the number and duration of diarrhea and vomiting episodes, body temperature, the severity of dehydration, and treatment modalities (rehydration therapy or hospitalization). The cases were classified into mild, moderate, or severe, as previously described (Supplement Table 1) [9, 10]. Samples were collected in clean, sterile, leakproof containers to be transported to the lab within 1 h [11], where each sample was divided into two sterile Eppendorf tubes to be stored at -20 C for ELISA tests and at -80 C for PCR tests.

**Table 1:** Demographic and clinical characteristics of total study children (n.92)

Variables		*N*.		%
Gender	Females	40		43.5
	Males	52		56.5
Age per months	≤ 6 months > 6–12 months > 12–18 month > 18–24 month > 24–30 month > 30–36 month	10 33 28 15 4 2		10.9 35.9 30.4 16.3 4.3 2.2
	Mean ± SD Median (range)	14.34 ± 6.98 13(4–36)		
Feeding pattern	Breastfeeding	30		32.6
	Bottle	44		47.8
	Mixed	3		3.3
	Weaning	15		16.3
Duration of diarrhea in days	Mean ± SD Median (range)	5.1 ± 2.73 5(1–14)		
Frequency of diarrhea time/day	Mean ± SD Median (range)	9.61 ± 4.16 8(4–20)		
Body temperature ℃	Mean ± SD Median (range)	39.06 ± 1 C 39(37-41.2)		
Duration of Vomiting in days	Mean ± SD Median (range)	3.32 ± 2.43 3(1–12)		
Frequency of vomit time per day	Mean ± SD Median (range)	7.49 ± 3.99 6(2–18)		
Vesikari clinical severity score	Severe	71	77.2	
	Moderate	21	22.8	

### Screening for RVA in stool samples

The RIDASCREEN^®^ Rotavirus ELISA kit (R-Biopharm AG, Germany) was used to screen for RVA in stool samples by detecting the RVA-specific VP6 antigen. The sandwich ELISA technique was utilized according to the manufacturer’s instructions.

### Viral RNA extraction from stool samples

Viral RNA was extracted from stool samples using the EASY-RED Total extraction kit (iNtRON Biotechnology, South Korea) following the manufacturer’s protocol. The extracted RNA was dissolved in 20–50 µl of RNase-free water and divided into three aliquots stored at -80 °C.

### Detection of RVA-specific VP6 coding gene in stool samples by nested RT-PCR

The RVA-specific VP6 coding gene was detected in stool samples using nested RT-PCR. Initially, a 379-bp segment of the VP6 coding gene was amplified by RT-PCR using forward primer VP6-F and reverse primer VP6-R, as described by Iturriza-Gomara et al. [12]. Subsequently, Following the method described by Gallimore et al. [13], nested PCR was performed on the previous RT-PCR products to amplify a 155-bp fragment. This was done using the forward primer VP6-NF and the reverse primer VP6-NR to enhance the detection sensitivity (Supplement Table 2).

**Table 2:** Performance of ELISA in relation to nested RT-PCR for diagnosis of RVA infections among children with acute gastroenteritis

		Nested RT-PCR		Sensitivity	Specificity		PPV	NPV	Accuracy
		+ve	-ve						
ELISA	+ve	22	0	91.7%		100%	100%	97.1%	97.8%
	-ve	2	68						

### Genotyping of the detected RVA in stool samples

The investigation into Common G Genotypes was achieved using a multiplex nested RT-PCR. The entire gene segment 9 coding for VP7 (1062 bp) was amplified via RT-PCR using forward primer Beg9 and reverse primer End9, as described by Gouvea et al. [14]. Subsequently, multiplex nested PCR amplification of the preceding RT-PCR products was performed to identify the common G genotypes. The primers used were forward primer RVG9 and G-type specific reverse primers aBT1 (G1 specific), aCT2 (G2 specific), aET3 (G3 specific), and aDT4 (G4 specific), with expected amplicon sizes of 749 bp, 652 bp, 374 bp, and 583 bp, respectively (Supplement Table 2). In addition, a monoplex nested RT-PCR amplification was performed on the previous RT-PCR products to detect the G9 genotype. This was done using the method outlined by Villena et al. [15], with the forward primer RVG9 and the reverse primer aFT9 (specific to G9). The expected size of the amplified DNA fragment was 306 bp (Supplement Table 2). The amplicons from the nested PCR were visualized using agarose gel electrophoresis.

The investigation into Common P Genotypes was achieved using multiplex semi-nested RT-PCR based on the methodology outlined by Gentsch et al. [16]. Initially, RT-PCR was used to amplify the VP8 fragment (876 bp) of gene segment 4 coding for VP4, using forward primer Con2 and reverse primer Con3. Subsequently, multiplex semi-nested PCR amplification of the preceding RT-PCR products was conducted using forward primer Con3 and p-type specific reverse primers 1T-1 (P[8] specific), 2T-1 (P[4] specific), and 3T-1 (P[6] specific), with expected amplicon sizes of 346 bp, 483 bp, and 267 bp, respectively (Supplement Table 2). Visualization of the amplicons from the nested PCR was achieved by agarose gel electrophoresis.

### Statistical analysis

All data were gathered, organized, and analyzed using IBM Corp.‘s IBM SPSS Statistics software, Version 23.0 (IBM Corp, 2015, Armonk, NY). Quantitative data were represented as mean ± SD and median (range), while qualitative data were depicted as numbers and percentages. The Mann-Whitney U test was used to compare two non-normally distributed variable groups. The Chi-square test or Fisher exact test, as appropriate, was employed to compare percentages of categorical variables. All statistical tests were two-tailed. Statistical significance was determined by a p-value ≤ 0.05; a p-value > 0.05 indicated statistical insignificance. Backward logistic regression was applied, beginning with all significant variables and eliminating variables from the regression model at each step to find a model that best explains the data.

## Results

Demographic and clinical attributes of 92 children with acute gastroenteritis included in the study are displayed in Table 1.

**Fig. 1:**
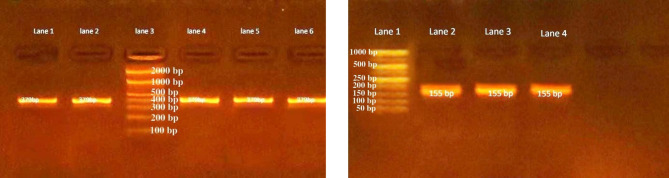
Agarose gel electrophoresis to visualize the products of nested RT-PCR for detection of RVA VP6 coding gene. RT-PCR (first reaction) was performed to amplify a 379-bp region of VP6 coding gene followed by nested PCR (second reaction) to amplify 155 bp fragment to increase detection sensitivity

Among 92 stool samples included in this study, RVA was detected at a rate of 24% (22 /92) by ELISA and 26.1% (24 /92) using VP6 nested RT-PCR (Fig. 1).

Comparing the results of ELISA for VP6 antigen to that of nested RT-PCR of VP6 coding gene as a gold standard, ELISA showed a sensitivity of 91.7%, a specificity of 100%, and 97.8% accuracy for diagnosis of RVA infections among children with acute gastroenteritis (Table 2).

**Fig. 2:**
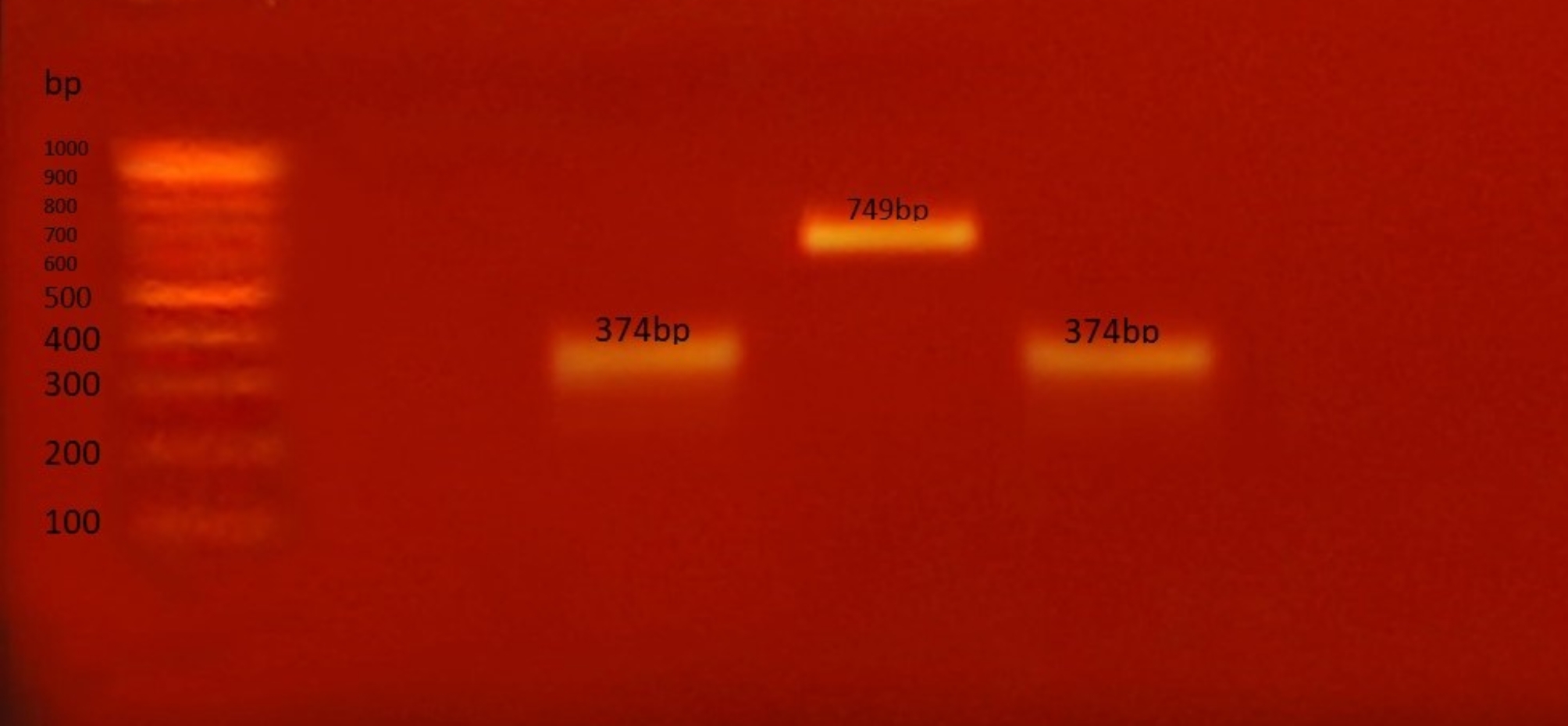
Agarose gel electrophoresis to visualize the products of multiplex nested RT‑PCR to detect common G Genotypes. Lanes 3 and 5 show G3 (374 bp), lane 4 shows G1 (749 bp), while G2, G4, G9 types were not detected in any of the lanes. The ladder used is 100 bp

In the present study, the prevalence of G types was examined. G3 was the most frequently detected type, accounting for 37.5% of the samples, followed by G1 at 12.5%. However, 50% of the samples could not be typed. G2, G4, G9 types were not detected in any samples (Fig. 2).

**Fig. 3:**
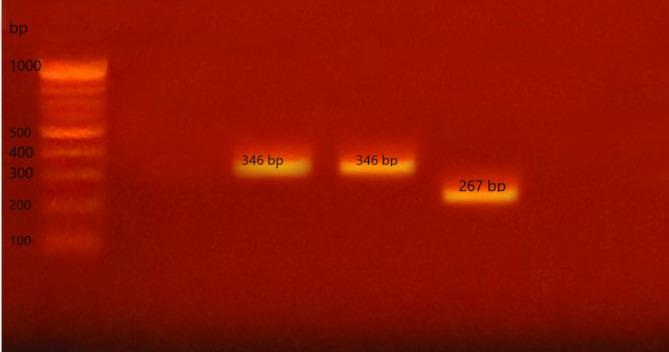
Agarose gel electrophoresis to visualize the products of multiplex nested RT‑PCR to detect common P Genotypes. Lanes 3 and 4 show P[8] (346 bp), lane 5 shows P[6] (267 bp) while P[4] was not detected in any lane. The ladder used is 100 bp

For P types, P[8](41.7%) was the most frequently detected type followed by P[6] (8.2%), 50% of samples could not be typed, and P[4] was not detected in any of the samples (Fig. 3).

The predominant combined genotype observed was G3P[8], representing 16.7%, followed by both G1P[8] and G3P[6] representing 8.3% each. Meanwhile, 4.1% was G1P[untypeable], 12.5% was G3P[untypeable], 16.7% was G untypeable P[8]. However, only 33.3% were untypeable for both G and P types (Table 3).

**Table 3:** Genotyping of the detected RVA (n.24)

RVA genotyping		*N*	%
G type	G1	3	12.5
	G3	9	37.5
	G untypeable	12	50
P type	P[6]	2	8.2
	P[8]	10	41.7
	p[untypeable]	12	50
Combined G and P type	G1P[8]	2	8.3
	G3P[6]	2	8.3
	G3P[8]	4	16.7
	G1P[untypeable]	1	4.1
	G3P[untypeable]	3	12.5
	G untypeable P[8]	4	16.7
	G untypeable P[untypeable]	8	33.3

The comparison between RVA positive and negative cases regarding demographic and clinical data and seasonal variation is presented in Table 4. RVA-positive samples were significantly associated with younger aged children (11.7 ± 5.69 vs. 15.26 ± 7.19 month, *p* = 0.026) with infection peaked at the age of 6–12 m (*P* = 0.052). RVA infection was significantly associated with bottle-feeding (*p* = 0.033). Significant seasonal variation was also reported, with the highest rates of RVA-positive cases occurring in cooler seasons (*p* = 0.0001). Rotaviral infections were significantly linked to more frequent episodes of diarrhea (10.87 ± 3.63 times/day) and vomiting (8.79 ± 3.57 times/day) per day (*p* = 0.013 and *p* = 0.011, respectively). Finally, severe Vesikari clinical score was significantly more common in children with RVA gastroenteritis (91.7% vs. 72.1%, *p* = 0.049).

**Table 4:** Demographic and clinical characteristics of RVA positive cases compared to negative cases

Variable		Positive RVA *N*.24	Negative RVA *N*.68	U/ X^2^	*P* value
Age in months	Mean ± SD	11.7 ± 5.69	15.26 ± 7.19	2.22	0.026*
	Median (range)	10(5–26)	15(4–36)		
Age group	≤ 6 months	4(40.0)	6(60.0)	***X*** ^***2***^ 10.97	0.052
	6–12 months	14(42.4)	19(57.6)		
	> 12–18 month	3(10.7)	25(89.3)		
	> 18–24	2(13.3)	13(86.7)		
	> 24–30 month	1(25.0)	3(75.0)		
	> 30–36 month	0.0	2(100.0)		
Gender	Male	12 (23.1)	40 (76.9)	***X*** ^***2***^ 0.562	0.453
	Female	12(30.0)	28(70.0)		
Feeding pattern	Breastfeeding	4(13.3)	26(86.7)	***X*** ^***2***^ 8.76	0.033*
	Bottle	16(36.4)	28(63.3)		
	Mixed	2(66.7)	1(33.3)		
	Weaned	2(13.3)	13(86.7)		
Season	Winter season	11(68.8)	5(31.2)	***X*** ^***2***^ 33.6	0.0001*
	Autumn	10(50.0)	10(50.0)		
	Summer season	2(4.3)	44(95.7)		
	Spring season	1(10)	9(90)		
Duration of diarrhea in days	Mean ± SD	4.46 ± 1.61	5.34 ± 3	1.009	0.313
	Median (range)	5(2–9)	5(1–14)		
Frequency of diarrhea time/day	Mean ± SD	10.87 ± 3.63	9.16 ± 4.27	2.486	0.013*
	Median (range)	10(4–20)	8(4–20)		
Body temperature℃	Mean ± SD	38.59 ± 0.89	39.23 ± 0.99	2.837	0.005*
	Median (range)	38.6(37-40.6)	39.4(37-41.2)		
Duration of Vomiting in days	Mean ± SD	2.62 ± 1.44	3.56 ± 2.66	1.231	0.218
	Median (range)	3(1–7)	3(1–12)		
Frequency of vomit time/day	Mean ± SD	8.79 ± 3.57	7.03 ± 4.05	2.544	0.011*
	Median (range)	8(2–18)	6(2–18)		
Vesikari clinical severity score	Severe	22(91.7)	49(72.1)	3.87	0.049*
	Moderate	2(8.3)	19(27.9)		

(p1 = breast feed versus bottle feed *p* = 0.028), (p2 = breast feed versus mixed feed *p* = 0.16), (p3 breast feed versus weaned *p* = 0.99)

(Comparison of summer versus Winter *p* = 0.00001), (comparison of summer versus autumn *p* = 0.0001), (comparison of summer versus spring *p* = 0.99)

Chi square test (x^2^), Mann-Whitney (U) * *p*:< 0.05 significant, *p*:≥0.05 no significant

The backward logistic regression was used to examine significant variables associated with RVA infection. It was found that older age was the only significant protective factor from RVA infection (*P* < 0.05) (Table 5).

**Table 5:** Logistic regression for predicting RVA infection in children (n.92)

Predictor	Sig.	Exp (B)	95% C.I for EXP(B)	
			Lower	Upper
**Age per months**	0.036*	0.915	0.842	0.994

Exp(β) = the odds ratios for the predictors, CI = Confidence interval, *p < 0.05 significant predictors

## Discussion

Group A rotavirus is the primary cause of infantile diarrhea worldwide, accounting for around 20% of deaths from diarrhea in children under the age of five. The impact is particularly significant in low-income countries and regions that do not have comprehensive RVA vaccination programs. These areas experience the highest burden of RVA-related diarrhea [17, 18]. Although there have been significant decreases in the global burden of rotavirus over the last three decades, the prevalence of rotavirus remains consistently high in regions such as Africa, Oceania, and South Asia [8].

Adequate understanding and data concerning the disease’s local prevalence, patterns, and age demographics are crucial for policymakers to assess the feasibility of including an rotavirus vaccine in their vaccination schemes. Furthermore, continuous monitoring of rotavirus genotypes is necessary to assess the potential coverage of prevalent genotypes by vaccines [19].

Among 92 stool samples collected through the study period from hospitalized children with acute gastroenteritis, RVA was detected at a rate of 24% by ELISA and 26.1% by VP6 nested RT-PCR. This indicates a higher sensitivity of the molecular techniques compared to ELISA. ELISA typically has a limited timeframe of approximately one week after the start of illness to identify viral shedding. However, RT-PCR can detect viral genetic material for extended durations beyond this initial period [18]. Similar rates have been previously reported in Egypt. Matson et al. [20] in 2010 reported that 25.2% of their samples were identified as rotavirus-positive samples among hospitalized children with gastroenteritis by ELISA. Another study, including Egyptian hospitalized children below five years old, reported that 31% of samples were positive for rotavirus by ELISA [21]. In addition, El-Senousy et al. [22], in 2020 reported that 24.37% of stool samples collected from children with acute diarrhea were positive for RVA using nested RT-PCR. In contrast, a clinical study conducted in Egypt on diarrhea in children involving two hospitals found that 23% and 10% of cases were attributed to rotavirus-associated diarrhea [23]. Another study, including Egyptian children below five years of age from a primary health care center with acute diarrhea, reported that rotavirus was the most prevalent organism detected in 10.7% of cases [24]. A study analyzed stool samples from inpatient and outpatient children and found that 39.1% tested positive for RVA. The occurrence of RVA was higher in samples from inpatients (43.9%) compared to those from outpatients (29.9%) [25]. The lower detection rates in some studies could be attributed to the different settings of these studies (patients from a clinic or a primary healthcare center demonstrate lower rates compared to hospitalized patients). Differences in inpatient and outpatient rotavirus prevalence have been previously reported in the literature [7].

Monitoring the prevalence of strains has become crucial in nations considering the implementation of a rotavirus vaccination program. This is necessary to assess whether the circulating strains are compatible with the serotypes included in the vaccines. By 2006, two live attenuated oral rotavirus vaccines had been licensed and widely deployed in numerous countries: Rotateq^®^, a pentavalent vaccine containing five live human-bovine rotavirus reassortants representing G1-4P[5] and G6P[8] types, and Rotarix^®^, featuring a live, attenuated, monovalent G1P[8] human rotavirus strain [26]. Despite demonstrating significant efficacy in extensive trials conducted across various geographical areas [27, 28], these vaccines were found to be comparatively less effective in African children and did not offer protection against all locally existing rotavirus G and P genotypes [29, 30]. Many countries worldwide have implemented rotavirus vaccination as recommended by the World Health Organization (WHO). Unfortunately, Egypt has not yet implemented rotavirus vaccination in the National immunization programs. Consequently, the vaccine is only available in private facilities [7, 31], and none of the children included in our study received rotavirus vaccines.

According to the Rotavirus Classification Working Group, a total of 42 G genotypes and 58 P genotypes have been documented [4]. Among these, the most commonly observed G types in human infections are G1, G2, G3, G4, and G9, while the prevalent P types are P[4], P[6], and P[8] [32]. Prevalent combinations of G and P genotypes include G1P[8], G2P[4], G3P[8], G4P[8], and G9P [8] [33].

In the present study, among the detected G types, G3 was the most common (37.5%), followed by G1 (12.5%), while 50% of the samples could not be typed. G2, G4, and G9 were not detected in any samples. For P types, P[8] was the most predominant (41.7%), followed by P[6] (8.2%), whereas 50% of the samples could not be typed, and P[4] was not detected in any of the samples. The most predominant combined genotype was G3P[8] (16.7%), followed by G1P[8] and G3P[6] (8.3% each). Additionally, 4.1% of the samples were G1P[untypeable], 12.5% were G3P[untypeable], 16.7% were G untypeable P[8], and 33.3% were untypeable for both G and P.

A comprehensive review and analysis of multiple studies examining viruses associated with acute gastroenteritis in African children under 5 years old found that the most common rotavirus genotype was G1P[8], which accounted for 39% of cases. Other prevalent genotypes included G3P[8] at 11.7%, G9P[8] at 8.7%, and G2P[4] at 7.1%. However, the analysis also identified some less common or unusual rotavirus genotypes, such as G3P[6] (2.7%), G8P[6] (1.7%), G1P[6] (1.5%), G10P[8] (0.9%), G8P[4] (0.5%), and G4P[8] (0.4%), circulating among the African pediatric population with acute gastroenteritis [34].

Previous investigations in Egypt have shown genotypic variability in rotaviruses over time. From 2000 to 2002, a study conducted on diarrhea in Egyptian children found that the most common strains were G1P[8], G2P[4], and G4P[8], accounting for 82.4% of the collected rotavirus strains. Additionally, G9 was detected in 5.3% of the samples [20]. Another study from 2011 to 2012 in Cairo identified the predominant genotypes as G3P[8] (37.7%) and G1P[8] (19.5%), with additional detection of uncommon genotypes such as G1P[6], G9P[6], G8P[14], and G12P[6] [25]. A study in Mansoura from 2010 to 2012 reported that G1P[8], G9P[8], and G3P[8] were the most common genotypes, presenting 62.3% of rotavirus gastroenteritis cases [35]. Another study conducted in Cairo from 2015 to 2016 found that the prevalent genotypes were G1P[8], G3P[8], and G1P[4], with G1P[8] being the most prevalent (29.7%) followed by G3P[8] (27.0%) [21].

In a study by El-Senousy et al. [22] in 2020, the most dominant G genotype observed was G1 (26%), followed by G3 (20.40%). G2 and G4 genotypes were not detected in their study, while G9 was found in 12.40% of the samples. A significant proportion of specimens (41.20%) remained untyped for the G genotype. Regarding P genotypes, P[4] was the most prevalent (40.00%), followed by P[8] (22.80%) and P[6] (19.60%).

Consistent global monitoring of rotavirus is essential to track the distribution of genotypes and detect the appearance and dissemination of new strains that may not be protected by current rotavirus vaccines. Assessing the efficacy and success of vaccination efforts is crucial [22, 36, 37].

The present study found a significant association between RVA-positive samples and younger children (11.7 ± 5.69 vs. 15.26 ± 7.19 months, *p* = 0.026). Furthermore, RVA infection peaked at the age of 6–12 m (*P* = 0.052). These findings support previous studies on rotaviral infections globally, indicating that infants under six months old were partially protected from infection by maternal antibodies, while those above the age of 18 months seemed to have acquired adequate immunity due to previous infections [22, 38].

The current study showed that RVA infection was significantly associated with bottle feeding (*p* = 0.033). Breastfeeding is essential for the development of an effective gut immune system in infants. It reduces the risk of acquiring gut diseases due to the presence of lactoferrin, maternal antibodies, and secretory immunoglobulin A (IgA), which provide protection against pathogens and complement the function of the immune system in infants [39, 40]. Moreover, introducing complementary food prior to the completion of the initial six months heightens the vulnerability to contamination, particularly in underdeveloped regions where access to clean drinking water and fundamental sanitation facilities is limited [41].

The present study revealed a notable seasonal fluctuation in the prevalence of RVA-positive cases, with the most elevated rates occurring during colder months (*p* = 0.0001). This pattern was also reported in other temperate climates [42] as well as in Egypt [22, 23, 25, 35]. Conversely, a separate study indicated a higher occurrence of rotavirus infections in the warmer months. This finding implies that the pattern of seasonality could differ from one year to another [24].

The current study found a strong correlation between RVA infections and frequent episodes of diarrhea (10.87 ± 3.63 times per day] and recurrent episodes of vomiting (8.79 ± 3.57 times per day] (*p* = 0.013 and *p* = 0.011, respectively]. Prior suggestions indicate that although rotavirus may result in fewer occurrences than other enteropathogens, it tends to cause a higher proportion of severe cases. Rotavirus is more commonly linked to symptoms such as a sudden start, frequent watery stools, repeated vomiting, dehydration, and the need for hospitalization, compared to other intestinal pathogens [23].

In the present study, using the Vesikari score to assess the severity of the diarrheal disorder, most diarrhea cases (77.2%] had severe Vesikari clinical scores while 22.8% had moderate scores. Similar results were reported by [43], suggesting that all the children with gastroenteritis included in the study were hospitalized. Severe scores were significantly more common in children with RVA gastroenteritis (91.7% vs. 72.1%, *p* = 0.049]. Saudi et al. [34] reported that children with rotavirus infection significantly increased the frequency of reported clinical manifestations, fever, vomiting, and dehydration. Nevertheless, the study did not use the Vesikari score. Mohakud et al. [43] reported that among those who tested positive for rotavirus, 74.39% had a severe score, 17.07% had a moderate score, 6.10% had a very severe score, and only 2.44% had a mild score. However, there was no significant difference between the positive and negative groups.

**In conclusion**, this hospital-based cross-sectional study highlights the significant impact of RVA infection as a common cause of acute gastroenteritis among children in our area. It also reveals the variety of RVA genotypes locally existing RVA genotypes and the presence of common G and P untypeable strains. These findings highlight the significance of incorporating the rotavirus vaccine into Egyptian national immunization programs while also maintaining ongoing monitoring of strains to assess any potential shifts in the epidemiology of rotavirus gastroenteritis. This will also allow for the evaluation of vaccine effectiveness against the prevalent genotypes in Egypt.

### Study limitations

The study’s limited sample size and absence of multiple study sites represent limitations within this study. Therefore, it is necessary to continuously monitor diarrheal pathogens in Egypt in order to assess the impact of the disease and aid in developing well-informed strategies for preventing rotavirus gastroenteritis among children.

## Electronic supplementary material

Below is the link to the electronic supplementary material.

Supplementary Material 1

Supplementary Material 2

Supplementary Material 3

Supplementary Material 4

Supplementary Material 5

## Data Availability

No datasets were generated or analysed during the current study.
