# Supplementary material for: Frequency and genotyping of group A rotavirus among Egyptian children with acute gastroenteritis: a hospital-based cross-sectional study
Source: Virol J. 2024 Sep 30;21:238. doi: 10.1186/s12985-024-02495-8 (PMC11443952; doi:10.1186/s12985-024-02495-8)
Supplement: Supplementary file 1 — Supplementary Material 1 [file 12985_2024_2495_MOESM1_ESM.docx]

**Frequency and Genotyping of Group A Rotavirus among Egyptian Children with Acute Gastroenteritis: A Hospital-based Cross-sectional Study**

***Ensaf Abd-elgawad Azazy ^a^, Rania M Amer ^a^, Ghada Mohammed Abdellatif ^b^,*** ***Hala Adel Abd-elmoneim^a^, Doaa Alhussein Abo-alella ^a*^.***

a Medical Microbiology and Immunology Department - Faculty of Medicine - Zagazig University - Elsharkia Governorate- Egypt.

b Pediatrics Department - Faculty of Medicine - Zagazig University - Elsharkia Governorate - Egypt.

*Corresponding author: [**DAAboelelaa@medicine.zu.edu.eg**](mailto:DAAboelelaa@medicine.zu.edu.eg)

ORCID ID: 0000-0002-2145-8817

**Supplement table 1: Vesikari Scoring System [9,10]**

| **Parameter** | **1** | **2** | **3** |
| --- | --- | --- | --- |
| **Diarrhea** |  |  |  |
| **Maximum number stools per day** | **1–3** | **4–5** | **≥6** |
| **Diarrhea duration (day)** | **1–4** | **5** | **≥6** |
| **Vomiting** |  |  |  |
| **Maximum number per day** | **1** | **2–4** | **≥5** |
| **Vomiting duration (day)** | **1** | **2** | **≥3** |
| **Maximum body temperature (℃)** | **37.1–38.4** | **38.5–38.9** | **≥39.0** |
| **Severity of dehydration (%)** | **N/A** | **1–5** | **≥6** |
| **Treatment** | **Rehydration** | **Hospitalization** | **N/A** |
| **Severity rating scales** | **<7 (mild)** | **7–10 (moderate)** | **≥11(severe)** |

**Supplement table 2: Sequences of primers utilized in the study for nested RT-PCR assays and their expected product sizes.**

| **Name** | **Sequence** | **Expected product size Bp** | **Reference** |
| --- | --- | --- | --- |
| **VP6-F** | **5′-GACGGVGCRACTACATGG T-3′** | **379** | **[12]** |
| **VP6-R** | **5′- GTCCAATTCATNCCTGGT GG-3′** |  |  |
| **VP6-NF** | **5′-GCWAGAAATTTTGATACA-3′** | **155** | **[13]** |
| **VP6-NR** | **5′-GATTCACAAACTGCAGA-3′** |  |  |
| **Beg9** | **5′-GGC TTT AAA AGA GAG AAT TTC CGT CTG G-3′** | **1062** | **[14]** |
| **End9** | **5′- GGT CAC ATC ATA CAA TTC TAA TCT AAG-3′** |  |  |
| **RVG9** | **5′- GGT CAC ATC ATA CAA TTC T-3′** |  |  |
| **aBT1 (G1 specifc)** | **5′-CAA GTA CTC AAA TCA ATG ATG G-3′** | **749** |  |
| **aCT2 (G2 specifc)** | **5′-CAA TGA TAT TAA CAC ATT TTC TGT G-3′** | **652** |  |
| **aET3 (G3 specifc)** | **5′-CGT TTG AAG AAG TTG CAA CAG-3′** | **374** |  |
| **aDT4 (G4 specifc)** | **5′- CGT TTC TGG TGA GGA GTT G-3′** | **583** |  |
| **aFT9 (G9 specifc)** | **5′-CTA GAT GTA ACT ACA ACT AC-3′** | **306** | **[15]** |
| **Con2** | **5′- ATT TCG GAC CAT TTA TAA CC-3′** | **876 bp** | **[16]** |
| **Con3** | **5′-TGG CTT CGC CAT TTT ATA GAC A-3′** |  |  |
| **1T-1 (P[8] specifc)** | **5′-TCT ACT TGG ATA ACG TGC-3′** | **346** |  |
| **2T-1 (P[4] specifc)** | **5′-CTA TTG TTA GAG GTT AGA GTC-3′** | **483** |  |
| **3T-1 (P[6] specifc)** | **5′-TGT TGA TTA GTT GGA TTC AA-3′** | **267** |  |
