# Supplementary figures and images for: Frequency and genotyping of group A rotavirus among Egyptian children with acute gastroenteritis: a hospital-based cross-sectional study
Source: Virol J. 2024 Sep 30;21:238. doi: 10.1186/s12985-024-02495-8 (PMC11443952; doi:10.1186/s12985-024-02495-8)

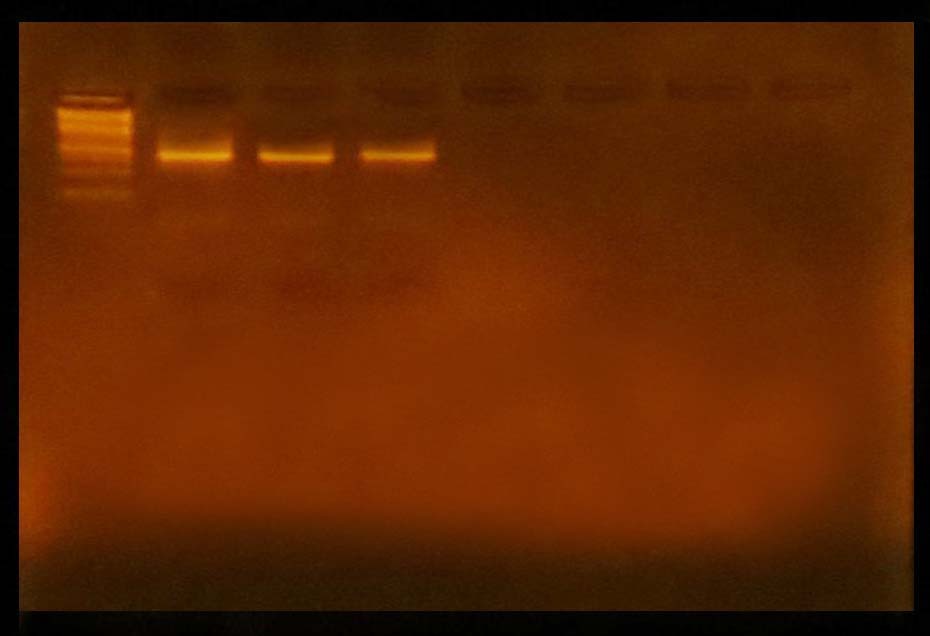

Supplement: Supplementary file 2 — Supplementary Material 2 [file 12985_2024_2495_MOESM2_ESM.jpeg]

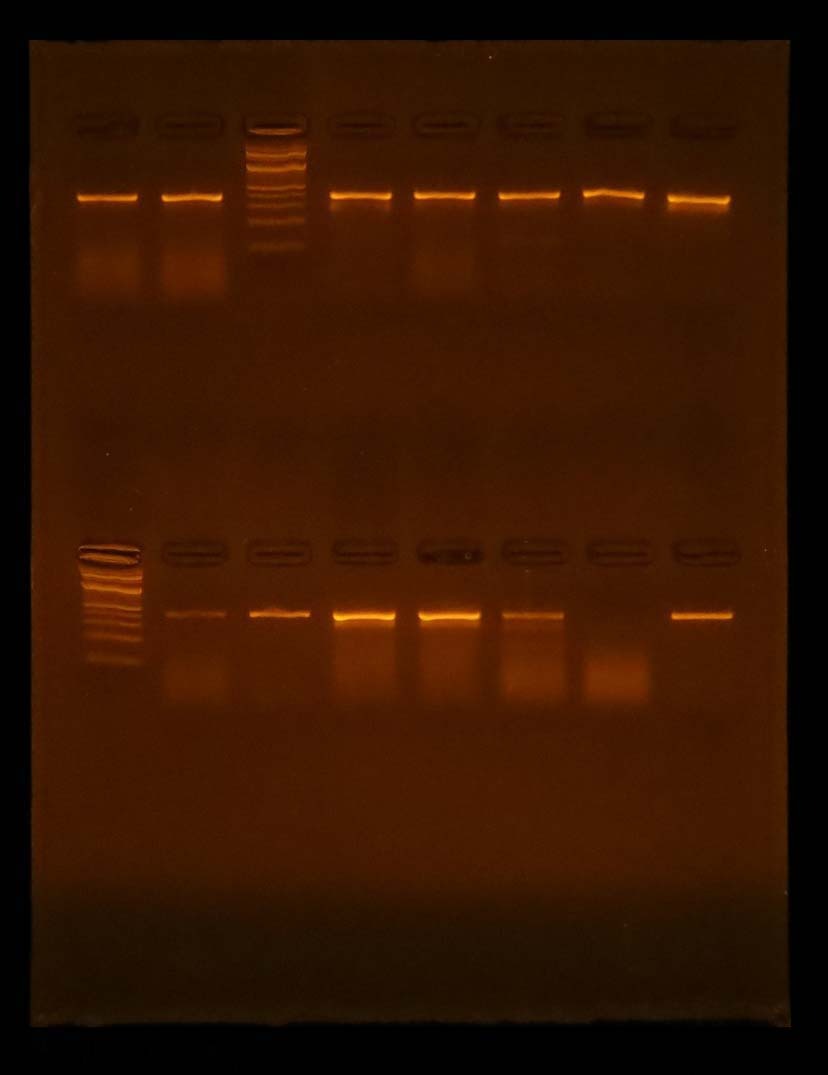

Supplement: Supplementary file 3 — Supplementary Material 3 [file 12985_2024_2495_MOESM3_ESM.jpeg]

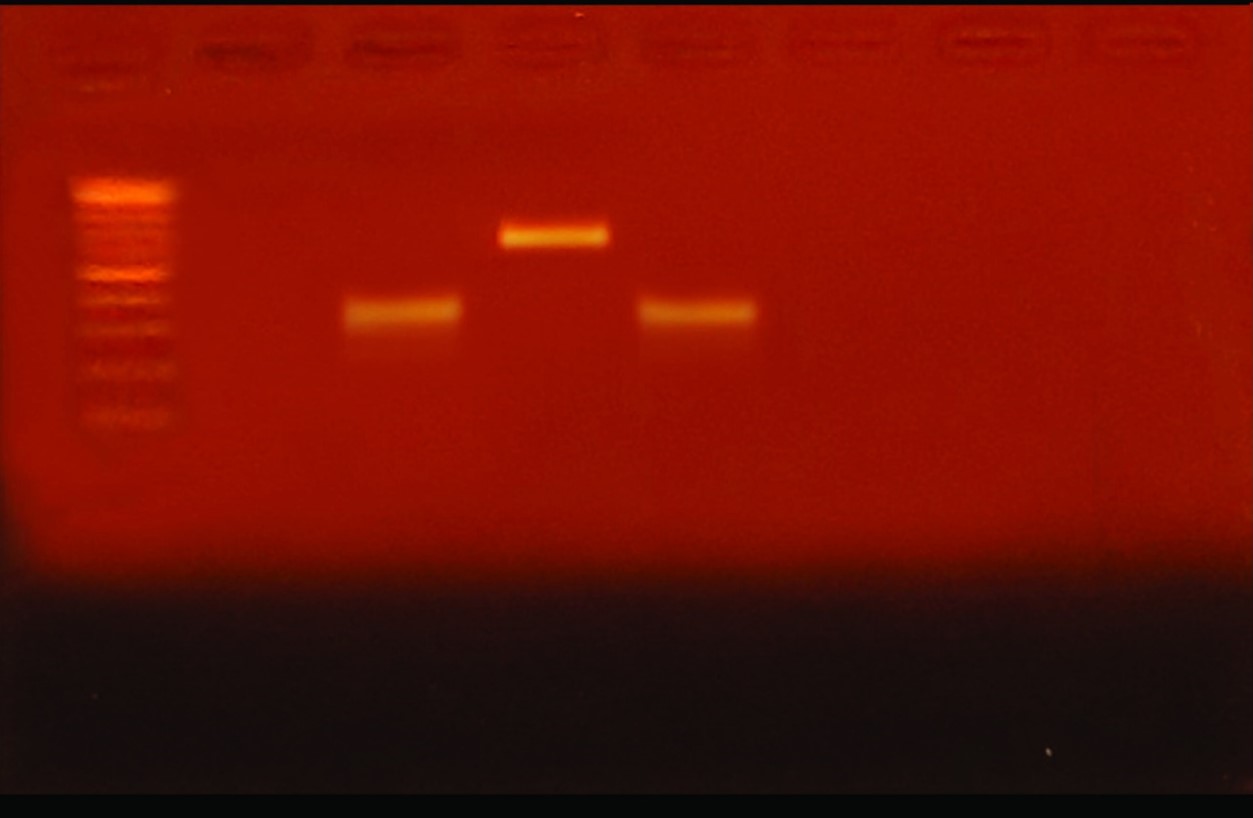

Supplement: Supplementary file 4 — Supplementary Material 4 [file 12985_2024_2495_MOESM4_ESM.jpeg]

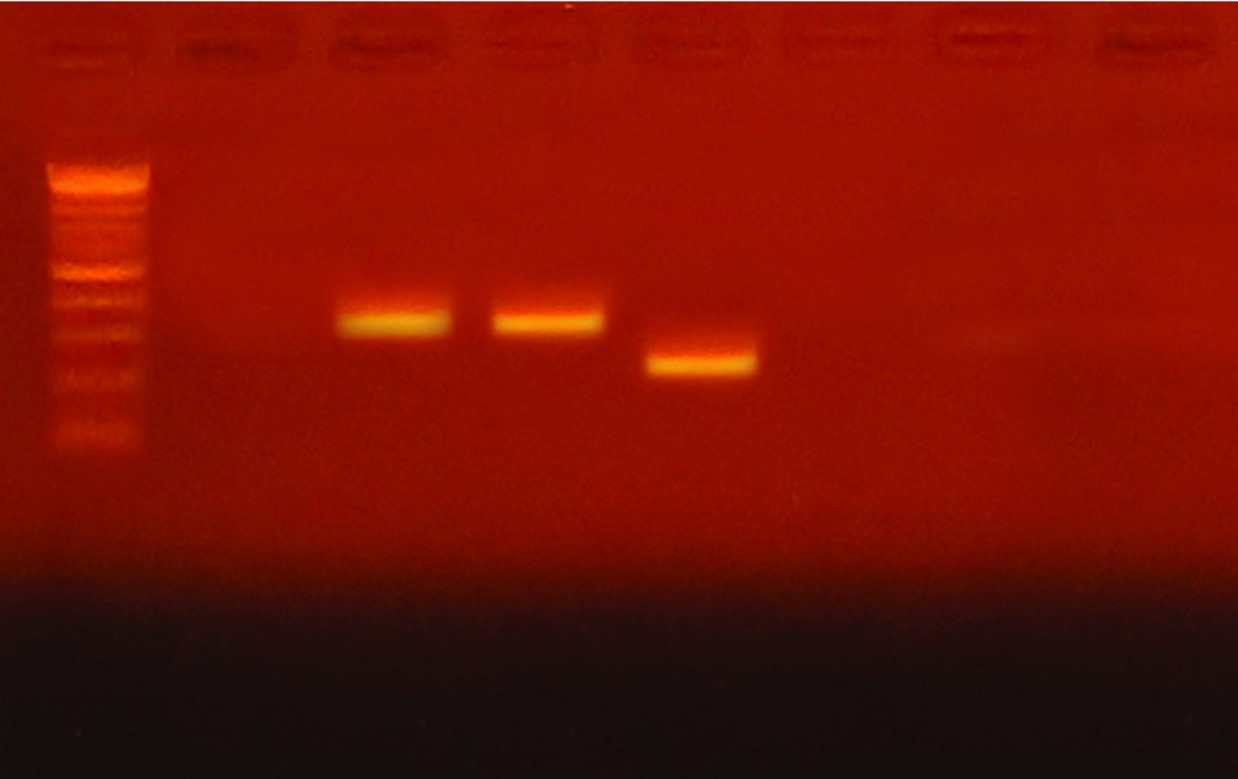

Supplement: Supplementary file 5 — Supplementary Material 5 [file 12985_2024_2495_MOESM5_ESM.jpeg]
